# Real-World Effectiveness of Different Nutraceutical Formulations on Pain Intensity of Subjects with Diabetic Peripheral Neuropathy: An Observational, Retrospective, Case–Control Study

**DOI:** 10.3390/biomedicines13061407

**Published:** 2025-06-08

**Authors:** Laura Armeli Grigio, Denisa Boci, Giacoma Di Vieste, Gianluca Cassanelli, Oscar Massimiano Epis, Alessandro Viadana, Federico Bertuzzi, Basilio Pintaudi

**Affiliations:** 1 Department of Endocrinology, Bicocca University, 20126 Milan, Italy; 2 Diabetes Unit, Niguarda Hospital, 20162 Milan, Italy; 3 Uriach Italy S.r.l., 20057 Assago, Italy; 4 Rheumatology Unit, Niguarda Hospital, 20162 Milan, Italy

**Keywords:** diabetic peripheral neuropathy, α-lipoic acid, N-acetyl cysteine, glutathione, pain intensity

## Abstract

**Background/Objectives**. Diabetic peripheral neuropathy is a debilitating disease-related complication with a significant impact on quality of life. Its management represents a therapeutic challenge. Antioxidant agents such as α-lipoic acid, N-acetyl cysteine, and glutatione may be useful treatment strategies. **Methods**. A real-world, observational, retrospective, case–control study involving consecutive subjects with type 2 diabetes with diabetic peripheral neuropathy was performed. Participants who were supplemented with three different formulations for 12 weeks (high-dose α-lipoic acid (800 mg); low-dose α-lipoic acid (100 mg) plus glutathione (200 mg) plus Vitamin D (800 IU); N-acetyl cysteine (600 mg) plus glutathione (200 mg) plus Vitamin D (800 IU)) were compared with a non-treated control group. Questionnaires aimed at investigating the degree of disability and quality of life were administered. The primary endpoint was the change in neuropathic pain intensity measured by the Numerical Rating Scale (NRS). **Results**. Among 750 consecutive screened subjects with type 2 diabetes, 98 (13%) had diabetic neuropathy (mean age 66.7 ± 7.6 years, diabetes duration 11.3 ± 6.7 years, HbA1c 8.1 ± 1.5%, 43.8% insulin-treated). When comparing the differences between treatment groups in the changes in individual questionnaire scores between baseline and follow-up, all three supplements showed significant reductions compared to the control group in the NRS scale scores. No side effects have been reported during the study. **Conclusions**. As well as lipoic acid, other substances with specific activity on the genesis of neuropathic pain, such as N-acetyl cysteine and glutathione, have proved effective in reducing the intensity of pain.

## 1. Introduction

Type 2 diabetes is a chronic disease with a huge socio-economic impact. It is characterized by a significant increase in direct and indirect healthcare costs, primarily resulting from the occurrence and evolution of its related complications [1]. Inadequate glycemic control is associated with a high risk of developing macrovascular (i.e., coronary artery disease, peripheral arteriopathy, cerebrovascular disease) and microvascular (i.e., retinopathy, nephropathy, and neuropathy) complications [2]. Of these, diabetic neuropathy in both the sensory-motor and autonomic forms represents one of the most debilitating complications, with a significant impact on the quality of life of people with diabetes and a considerable economic burden on healthcare systems [3]. Diabetic sensory-motor neuropathy is the most frequent form and manifests itself with symptoms such as pain and sensory changes (paresthesia, urent dysesthesia, hypoesthesia), most commonly affecting the lower limbs. Its global prevalence has been estimated in a study involving subjects from 14 different countries. The overall prevalence was 26.7%, with a considerable country variation [4]. Autonomic forms of neuropathy involving the autonomic nervous system are less common but equally serious, leading to dysfunctions such as orthostatic hypotension, impaired gastrointestinal motility, and urogenital dysfunction. Screening for diabetic neuropathy, especially in its sensory-motor form, is of great importance because it is associated with a 1.7-fold increased risk of lower-limb amputation in patients with a negative history of lower-limb ulceration, while in cases of subjects with a history of lower-limb ulcers, the risk increases 36-fold [5].

The treatment of diabetic neuropathy, according to the recommendations of the American Diabetes Association, requires a multitarget approach through both symptomatic and pathogenetic treatment. In fact, no treatment is effective unless glycol-metabolic compensation is achieved, with good control of arterial hypertension and dyslipidemia due to the high sensitivity of nerve fibers to free radicals and advanced glycation compounds that are produced in all these conditions [6]. The management of neuropathic pain represents a therapeutic challenge, as most analgesic drugs do not provide remission of symptoms [7]. Symptomatic treatment is aimed at reducing pain with a consequent relative improvement in quality of life and, in accordance with the recommendations of the American Academy of Neurology and the European Federation of Neurological Societies, mainly involves the use of anti-epileptic drugs and selective serotonin reuptake inhibitor antidepressants [8].

In the genesis of neuropathic pain, increased oxidative stress is recognized as one of the possible causes of nerve damage, inducing axonal degeneration and myelin degradation of nerve fibers [8]. According to this rationale, antioxidant agents may represent a useful treatment strategy for patients with diabetic neuropathy. Among these, α-lipoic acid has been proposed for the treatment of patients with peripheral nerve injury. The effectiveness of alpha-lipoic acid in clinical practice has been evaluated in several clinical studies conducted on patients with different forms of neuropathy: diabetic neuropathy, back pain secondary to disc herniation, and carpal tunnel syndrome [9,10]. It has demonstrated positive effects in reducing symptoms associated with diabetic neuropathy [10]. α-lipoic acid increases levels of glutathione, an endogenous antioxidant involved in protection from oxidative stress and in nutrient metabolism, without causing significant adverse reactions [11]. The characteristic presence of sulphydryl groups with antioxidant and anti-inflammatory activity can also be found in other substances, such as N-acetyl cysteine, which has been used in the treatment of neuropathic pain due to these properties [12].

The aim of the study was to evaluate the clinical effects of three different therapeutic formulations used as supplements in the case of diabetic sensorimotor neuropathy by comparing them with a control group that did not receive any specific therapy or supplementation but still had a diabetes-related neuropathic state.

## 2. Materials and Methods

This study was an observational, retrospective, case–control study. Data from subjects with type 2 diabetes mellitus, followed at the Diabetes Outpatient Clinic of Niguarda Hospital, Milan, in the period between December 2023 and October 2024, were analyzed. This study fell within the context of the diabetes care improvement program currently underway at our facility (approval no. 603-29092021, Ethics Committee Milan Area 3) and, in particular, addressed the evaluation of the efficacy of therapeutic strategies in the area of diabetic neuropathy complications. This study has been carried out in accordance with the Declaration of Helsinki. Informed consent for participation was obtained from all subjects involved in the study. Data from patients consecutively seen at the outpatient diabetes clinic who were prescribed supplementation for the treatment of neuropathy were analyzed. The subjects involved were those who tested positive on the Douleur Neuropathique 4 Questions (DN4) questionnaire, a well-validated questionnaire designed to screen for diabetic neuropathy [13]. The DN4 is a questionnaire consisting of ten questions divided into two sections. The first section includes seven questions related to the characteristics of pain and dysesthesia. The second section includes a brief objective examination with three clinical tests to assess the transmission of tactile and pain sensitivity. The questionnaire awards one point for each positive response, for a maximum total score of 10. A value of 4 or more suggests a probable neuropathic origin of the pain. The DN4 represents a valid screening tool for diabetic neuropathy, as it demonstrates, at the cut-off of 4, a sensitivity of 80% and a specificity of 91% for the diagnosis of neuropathic pain [14] and painful diabetic neuropathy [15]. We use this questionnaire as per standard care. Patients who tested positive on the questionnaire (i.e., DN4 score ≥4) were offered supplementation for the treatment of neuropathic pain.

Three therapeutic formulations were prescribed: (1) high-dose α-lipoic acid (800 mg) (Tiobec 800); (2) low-dose α-lipoic acid (100 mg), glutathione (200 mg), and Vitamin D (800 IU); (3) N-acetyl cysteine (600 mg), glutathione (200 mg), and Vitamin D (800 IU) (Tiobec). All the formulations were produced by Uriach Italy S.r.l., Assago Milanofiori Strada 1, Palazzo F6, 20057, Assago (MI). All the formulations used were available in clinical practice and had indications for use in cases of diabetic neuropathy. No selection of patients to be treated was made because three different diabetologists used different therapeutic approaches with different formulations for all the consecutive samples of their patients. This could lead to a different patient distribution among the study groups.

The effects of the supplementation were compared with a control group, represented by consecutive subjects who received neither medical therapy nor specific supplementation but who nevertheless presented a diabetes-related neuropathic disease. The inclusion criteria were as follows: aged between 18 and 80 years, an established diagnosis of diabetic neuropathy, and a neuropathic origin of pain. The exclusion criteria were as follows: an intake of substances contained in the products under study; a severe neurological motor deficit; antioxidants or anti-inflammatory medicine use; pregnancy or lactation. All clinical assessments, including those of major diabetologic relevance (i.e., glycemic compensation, disease-related complications, therapies), were taken before the start of treatment and after 12 weeks of therapy.

The primary endpoint of this study was the evaluation of the three therapeutic formulations on the intensity of neuropathic pain at 12 weeks after the start of treatment compared to baseline. The primary outcome of this study was the NRS (Numerical Rating Scales), which was used to assess perceived pain intensity [16]. The NRS is a numerical measure of pain intensity, ranging from 0 (no pain) to 10 (worst pain imaginable). The patient assigns a value based on his or her perception, allowing a subjective but reproducible assessment of the intensity of pain experienced by the patient at a given time. Due to its simplicity, rapidity, and versatility, the NRS is widely used in pain monitoring and the assessment of responses to analgesic treatments.

The secondary endpoints were the assessment of the three therapeutic formulations at 12 weeks after treatment on the degree of disability and quality-of-life parameters. For the assessment of these endpoints, questionnaires were administered both at the baseline and 12 weeks after the start of the therapy.

In particular, the following questionnaires were administered according to clinical practice as follows: the Brief Pain Inventory (BPI), the Problem Areas in Diabetes (PAID-5), the WHO-5, and the Self-Care Diabetes Activities Scale (SDSCA-6). The BPI is a scale used to measure the intensity of pain and interference with daily activities [17]. The PAID-5 measures the level of psychological distress experienced by people with diabetes due to the disease through assessment of the five main areas that can create psychological difficulties in subjects with diabetes (i.e., frustration with the disease, concern for the future, difficulty in self-management, feelings of loneliness, and the perception of being overwhelmed by the disease) [18]. The WHO-5 is a five-question questionnaire used to measure the psychological well-being of the individual by assessing the level of happiness and psychological satisfaction experienced by the individual in the last week [19]. The SDSCA-6 is a questionnaire that measures the level of compliance in the self-management of diabetes, specifically with regard to adherence to diet, self-monitoring of blood glucose, foot care, and taking hypoglycemic therapy [20].

Information was collected regarding demographic (age, sex), anthropometric (weight, height), lifestyle (smoking, potus, physical activity), clinical, and therapeutic variables. With regard to clinical variables, laboratory parameters (lipid profile, renal function, fasting glycemia, glycated hemoglobin, transaminases), data on screening for disease-related complications (nephropathy, retinopathy, macroangiopathy, diabetic foot), and comorbidities (heart disease, arterial hypertension, dyslipidemia) were considered. At the follow-up contact, the level of adherence to the suggested therapy was investigated by means of an anamnestic survey.

### Statistical Analysis

Data were expressed as means ± standard deviation for the continuous variables and percentages for categorical variables. The Kolmogorov–Smirnov test was used to test the normality of the distribution of continuous variables. Clinical and demographic characteristics were compared using the Student’s t-test for normally distributed data and the Mann–Whitney test for not normally distributed numerical data. Between-group comparisons were performed by using a one-way ANOVA test in the case of normally distributed continuous variables and by using a Kruskal–Wallis test in the case of not normally distributed continuous variables or ordinal variables. A sample of 30 subjects taking the supplement was estimated as sufficient to detect a 4-point difference in the NRS numerical scale between the group receiving supplementation and the control group at 12 weeks after the start of treatment (α: 0.05, β: 0.80). A *p*-value <0.05 was considered for statistical significance. Analyses were performed using SPSS version 21.0 (SPSS, Inc., Chicago, IL, USA).

## 3. Results

**Figure 1:**
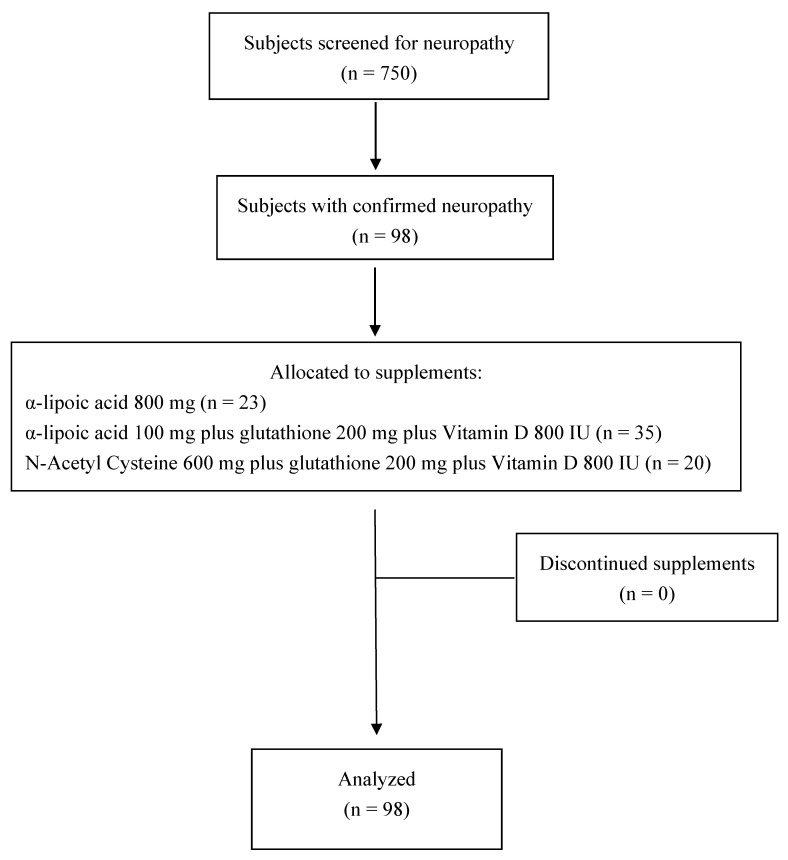
Flowchart of study participants.

A total of 750 consecutive subjects with type 2 diabetes mellitus were screened and administered the DN4 questionnaire. Of these subjects, 98 were found to have diabetic neuropathy, thus giving a prevalence of 13% in our population (Figure 1).

Overall, people with diabetic neuropathy had a mean age of 66.7 ± 7.6 years, a mean diabetes duration of 11.3 ± 6.7 years, and the mean HbA1c levels were 8.1 ± 1.5% (65 ± 7 mmol/mol); this documented a not well-compensated diabetes; in 43.8% of the cases, insulin was part of the treatment. The baseline clinical characteristics of the subjects with diabetic neuropathy according to the supplemented therapy are shown in Table 1. The groups showed some differences in certain characteristics at baseline, including smoking habits, alcohol consumption, physical activity, prevalence of arterial hypertension, body weight, and HDL cholesterol levels (Table 1). Among the hypoglycemic treatment patterns, there was no difference between the groups in the percentage of insulin use. Table 2 shows the questionnaire scores at both baseline and follow-up. When comparing the differences between treatment groups in the changes in individual questionnaire scores between baseline and follow-up, all three supplements showed significant reductions compared to the control group in the NRS scores (Table 3). No differences in NRS were detected when comparing group 2 vs. group 3 (*p* = 0.46) or vs. group 4 (*p* = 0.87) and group 3 vs. group 4 (*p* = 0.52). Subjects treated with the supplement made by N-acetyl cysteine, glutathione, and Vitamin D showed lower scores in variations in SDSCA-foot care and SDSCA-self-monitoring of blood glucose compared to the control group. No between-group differences in BPI, WHO-5, or PAID-5 scores were detected. No side effects have been reported during the study.

## 4. Discussion

### 4.1. Main Findings

In the context of real-world activity, our study made it possible to document the effectiveness of certain supplements used to control pain symptoms in diabetic neuropathy. Compared with subjects taking no medical therapy or supplementation, those taking supplementation had a significant benefit on the extent of perceived pain, as measured by a validated scale such as the NRS. In particular, all the tested supplements were equally effective in reducing pain.

### 4.2. Comparison with Existing Literature

Current therapy for the treatment of diabetic neuropathy involves the use of antioxidants or substances with neurotropic action in addition to the strict control of glyco-metabolic status and all cardiovascular risk factors. The inhibition of aldose reductase activity and improvement in microcirculation have also been counted among the possible interventions. Alpha-lipoic acid, a coenzyme involved in the tricarboxylic acid cycle, acts as an antioxidant and can improve distal nerve conduction.

A recent systematic review and meta-analysis of randomized clinical trials aimed to assess the effects of alpha-lipoic acid as a disease-modifying agent in people with diabetic peripheral neuropathy [21]. The primary outcome was a change in neuropathy symptoms expressed as changes in the Total Symptom Score at six months after randomization. The authors concluded that alpha-lipoic acid probably had little or no effect on neuropathy symptoms or adverse events at six months. The results were probably influenced by the fact that all the included studies were at high risk of attrition bias. In addition, no studies reported quality of life or complications associated with diabetic peripheral neuropathy.

In the case of any supplementation or pharmacological treatment, particular attention must be paid to safety aspects. The use of alpha-lipoic acid has been associated with the possible occurrence of a rare autoimmune syndrome characterized by spontaneous hypoglycemia known as Hirata syndrome. This syndrome was first described in 1970 by Yukimasa Hirata in Japan, the country in which the largest number of cases was subsequently recorded [22]. The syndrome is characterized by the presence of elevated plasma insulin levels without a concomitant increase in C-peptide endogenous anti-insulin antibodies in subjects not previously treated with insulin, as well as the frequent intake of substances containing sulphydryl groups, including lipoic acid. Hirata’s syndrome associated with the use of alpha-lipoic acid was described for the first time in Italy in six patients not previously treated with insulin but hospitalized for severe hypoglycemia. The hypoglycemic symptoms occurred between 30 and 120 days after taking lipoic acid at a dose of 600 mg, and the hypoglycemic episodes reduced when the supplement was discontinued. In addition to the administration of intravenous glucose solution, it had almost always been necessary to combine 25 mg/day of prednisone in successively graduated doses, both for immuno-suppressive and counter-regulatory effects. In all patients, their serum insulin values returned to normal within 3 months after discontinuation of lipoic acid, with complete remission of hypoglycemic symptoms [23].

Although reported in a very small proportion of cases, in order to avoid the occurrence of this syndrome, it may be useful to use other substances with adequate efficacy in treating diabetic neuropathy symptoms. One of these is certainly N-acetyl cysteine. Recently, the use of N-acetyl cysteine was compared to standard care in the context of a randomized, parallel, open-label, controlled clinical trial involving patients with diabetic peripheral neuropathy [24]. Glutathione peroxidase, nuclear factor erythoid-2 related factor, and tumor necrosis factor were measured at baseline and after 12 weeks to assess the antioxidant and anti-inflammatory properties. Specific questionnaires were administered in order to investigate symptoms and quality of life. The authors concluded that the supplementation of N-acetyl cysteine modulated inflammation by reducing TNF-alpha and increasing glutathione peroxidase and nuclear factor erythoid-2 related factor, improved pain scores, and improved quality of life [24]. N-Acetylcysteine’s long-term cardiovascular benefits as an antioxidant with endothelial protective properties were investigated regarding a Major Adverse Cardiovascular Events (MACE) risk in T2DM patients, focusing on its potential as an adjunctive therapy [25]. A population-based cohort study used data from Taiwan’s National Health Insurance Research Database and included 46.718 T2DM patients. After propensity score matching, N-acetylcysteine users showed a significantly lower incidence of MACE (41.74% vs. 46.87%, *p* < 0.0001) compared to no users. Adjusted Hazard Ratios (aHRs) indicated a consistent protective effect of N-acetyl cysteine against an overall MACE (aHR: 0.84; 95% CI: 0.81–0.86, *p* < 0.0001). In our study, the supplement containing N-acetyl cysteine was effective in reducing pain, equal to other supplements containing lipoic acid. Considering the absolute safety level, its strong anti-inflammatory properties, and probably its effect on other vascular patterning factors underlying predisposing or determining atherosclerotic vascular pathology in individuals with diabetes, N-acetyl cysteine can be considered of great clinical interest.

### 4.3. Implications for Clinical Practice

This study is part of an initiative aimed at improving current clinical practice. In the context of benchmarking activities, it is important and interesting to analyze the efficacy and safety of certain therapies or procedures for which there are no explicit guidelines or recommendations. Consequently, it is essential to identify both the sub-population of subjects with the greatest benefit and the therapies that can offer the best clinical advantages in terms of efficacy. It is precisely for this reason that our study represents a real-world experience with important practical clinical implications.

Real-world research is of paramount importance, as it allows us to analyze the efficacy and safety of therapies in the everyday clinical setting outside the strict selection criteria of randomized clinical trials. By monitoring patients in everyday conditions, this research provides more representative and applicable data, allowing a more accurate assessment of treatment impact, treatment adherence, and quality of life, all of which directly influence clinical decisions and improve health outcomes [26].

Having a greater awareness of the best formulation to take in the case of diabetic neuropathy makes it possible to address the choice of the best medication in accordance with the patient’s general clinical state. In addition to data on the extent of pain symptoms, our study offers the possibility of looking at aspects of quality of life and the impact that diabetic disease complicated by neuropathy can have on the person with diabetes.

### 4.4. Strengths and Limitations

The limitations of our study are certainly those related to its observational design. For this reason, it was not possible to report a cause–effect association between the types of supplementation used and the intensity of pain. Another limitation lies in the small sample size studied. However, our sample size proved to be sufficiently powered to demonstrate adequate statistical significance in the primary outcome of the study, which was pain. Therefore, the between-groups comparison did not consider a homogeneous number of patients, being the patients were distributed unequally between the study groups. The strengths of the study are the systematic assessment of neuropathy by means of a standardized instrument. This reduced the possibility of selection bias.

## 5. Conclusions

The real-world effectiveness of therapeutic formulations used as supplements in the case of diabetic sensorimotor neuropathy represents important scientific evidence. Diabetic peripheral neuropathy is a disease-related complication that requires a rapid diagnosis that must necessarily be followed by the beginning of effective drug therapy. Both the use of lipoic acid and the use of other substances with specific activity on the genesis of neuropathic pain, such as n-acetil-cysteine and glutathione, have proved effective in reducing the intensity of pain, the most important and debilitating symptom for the patient suffering from this complication. Future changes to nutraceutical formulations are probably required in order to better mix their antioxidant and anti-inflammatory activities. Further studies with larger case series and a randomized controlled design are probably needed to provide more robust clinical data.

## Figures and Tables

**Table 1:** Clinical characteristics of the studied subjects.

	Group 1	Group 2	Group 3	Group 4	*p*
N	20	23	35	20	
Males (%)	40.0	69.6	57.1	75.0	0.10
Age (years)	61.8 ± 7.4	62.7 ± 8.5	67.7 ± 7.1	66.5 ± 5.1	0.60
Diabetes duration (years)	14.6 ± 5.5	10 ± 3.7	13.4 ± 5.9	10.8 ± 8.7	0.11
Smokers (%)	25.0	17.4	14.3	25.0	0.001
Alcohol intake (%)	25.0	8.7	14.3	0.0	0.006
Physical activity (%)	37.0	21.7	14.3	50.0	0.03
Family history of diabetes (%)	57.1	43.5	57.1	50.0	0.77
Heart disease (%)	54.5	21.7	28.6	50.0	0.10
Hypertension (%)	63.6	39.1	85.7	75.0	0.002
Dyslipidemia (%)	50.0	43.5	71.4	75.0	0.07
Weight (kg)	74.5 ± 21.3	85.4 ± 16.0	70.6 ± 7.1	65.3 ± 14.9	<0.001
Height (cm)	160.6 ± 9.2	167.5 ± 7.5	169.4 ± 7.8	166.7 ± 6.4	0.02
Systolic blood pressure (mmHg)	125 ± 10	127 ± 11	126 ± 9	131 ± 14	0.38
Diastolic blood pressure (mmHg)	75 ± 6	75 ± 6	77 ± 5	77 ± 8	0.60
Serum creatinine (mg/dL)	1.0 ± 0.3	0.9 ± 0.2	1.0 ± 0.3	0.9 ± 0.1	0.77
Urinary albumin (mg/L)	47.1 ± 87.6	8.4 ± 15.3	2.9 ± 4.5	0.9 ± 0.1	<0.001
Glycated Hemoglobin (%)	7.7 ± 1.1	8.2 ± 1.0	8.4 ± 2.0	7.6 ± 1.0	0.14
Fasting blood glucose (mg/dL)	163.6 ± 57.0	164.8 ± 40.2	169.4 ± 42.0	142.0 ± 31.8	0.13
Serum total cholesterol (mg/dL)	151.2 ± 28.6	160.2 ± 38.5	155.7 ± 40.6	161.7 ± 30.8	0.91
Serum LDL cholesterol (mg/dL)	92.5 ± 17.7	81.2 ± 28.9	79.9 ± 26.9	78.4 ± 30.0	0.92
Serum HDL cholesterol (mg/dL)	38.3 ± 5.5	54.2 ± 10.8	50.9 ± 10.0	60.0 ± 10.0	0.01
Triglycerides (mg/dL)	147.7 ± 71.8	124.8 ± 51.6	128.1 ± 37.9	113.2 ± 68.5	0.59
AST (mg/dL)	24.0 ± 4.2	21.8 ± 7.2	23.7 ± 7.5	18.0 ± 5.0	0.03
ALT (mg/dL)	26.0 ± 1.4	24.5 ± 16.1	28.3 ± 19.0	17.5 ± 5.6	0.12
Carotid Artery Stenosis (%)	10.0	29.4	42.9	25.0	0.16
Retinopathy (%)	18.2	5.9	14.3	25.0	0.45
Diabetic Foot (%)	9.1	5.9	0.0	25.0	0.01
Metformin (%)	40.0	60.9	71.4	50.0	0.12
Pioglitazone (%)	0	0.0	0.0	0.0	NA
Sulfonylurea (%)	25.0	0.0	0.0	0.0	0.001
Acarbose (%)	15.0	0.0	0.0	0.0	0.007
DPP4-i (%)	10.0	21.7	28.6	0.0	0.04
SGLT2-i (%)	35.0	43.5	57.1	50.0	0.43
GLP1-RA (%)	35.0	30.4	14.3	50.0	0.04
Insulin (%)	35.0	47.6	57.1	25.0	0.10

Group 1: no treatment (control group); group 2 treated with α-lipoic acid (800 mg); group 3 treated with α-lipoic acid (100 mg), glutathione (200 mg), and Vitamin D (800 IU); group 4 treated with N-acetyl cysteine (600 mg), glutathione (200 mg), and Vitamin D (800 IU). Between-group comparisons were performed by using a one-way ANOVA test in the case of normally distributed continuous variables and by using the Kruskal–Wallis test in the case of not normally distributed continuous variables or ordinal variables.

**Table 2:** Questionnaire scores at baseline and at follow-up.

	Group 1	Group 2	Group 3	Group 4	*p* Group 1 vs. Group 2	*p* Group 1 vs. Group 3	*p* Group 1 vs. Group 4
N	20	23	35	20			
Baseline DN4	6.1 ± 1.4	6.0 ± 1.5	6.3 ± 1.8	5.7 ± 0.4	0.82	0.69	0.0
Follow-up DN4	3.9 ± 2.3	3.8 ± 2.3	4.1 ± 2.4	3.5 ± 2.1	0.96	0.71	0.63
Baseline BPI severity score	17.6 ± 6.4	18.2 ± 6.1	19.1 ± 6.9	16.2 ± 3.9	0.78	0.43	0.41
Follow-up BPI severity score	12.5 ± 8.0	13.0 ± 8.2	15.3 ± 7.4	8.2 ± 7.6	0.83	0.20	0.09
Baseline BPI interference score	27.8 ± 12.8	28.5 ± 12.1	32.4 ± 11.5	21.0 ± 9.8	0.87	0.18	0.06
Follow-up BPI interference score	15.3 ± 11.3	16.3 ± 12.2	20.3 ± 11.1	8.2 ± 10.1	0.79	0.12	0.04
Baseline NRS	3.3 ± 2.9	3.6 ± 3.3	3.6 ± 3.7	3.5 ± 3.1	0.71	0.78	0.95
Follow-up NRS	5.5 ± 1.8	2.0 ± 2.5	1.3 ± 1.8	1.5 ± 2.1	<0.0001	<0.0001	<0.0001
Baseline WHO-5	13.6 ± 5.5	14.1 ± 5.4	12.4 ± 5.5	17.2 ± 3.9	0.77	0.45	0.02
Follow-up WHO-5	14.8 ± 6.3	13.6 ± 6.1	12.1 ± 6.1	17.2 ± 4.0	0.53	0.12	0.16
Baseline PAID-5	4.1 ± 2.8	4.6 ± 3.4	3.4 ± 2.5	5.7 ± 3.7	0.67	0.33	0.13
Follow-up PAID-5	5.4 ± 4.3	5.3 ± 3.7	5.0 ± 3.6	5.5 ± 4.0	0.88	0.68	0.97
SDSCA-6							
Baseline nutrition	4.4 ± 2.2	7.4 ± 2.3	4.9 ± 1.5	3.5 ± 3.1	0.99	0.36	0.30
Follow-up nutrition	5.5 ± 1.3	5.5 ± 1.1	5.0 ± 1.1	6.2 ± 0.4	0.95	0.50	0.02
Baseline physical activity	4.5 ± 2.6	4.7 ± 2.6	3.4 ± 2.5	7.0 ± 0.0	0.82	0.13	<0.0001
Follow-up physical activity	4.6 ± 2.7	4.8 ± 2.6	3.6 ± 2.7	6.7 ± 0.4	0.87	0.99	0.002
Baseline SMBG	4.1 ± 3.2	4.1 ± 3.0	3.7 ± 3.3	5.0 ± 2.2	0.95	0.64	0.04
Follow-up SMBG	4.9 ± 2.7	6.4 ± 1.5	4.1 ± 2.6	4.7 ± 2.9	0.98	0.31	0.87
Baseline SMBG adherence	4.1 ± 3.2	4.1 ± 3.0	3.1 ± 3.3	7.0 ± 0.0	0.87	0.25	0.16
Follow-up SMBG adherence	4.9 ± 2.7	6.4 ± 1.5	6.7 ± 0.7	5.7 ± 2.0	0.98	0.31	0.29
Baseline diabetic foot care	3.0 ± 2.9	3.7 ± 3.0	2.3 ± 2.8	6.2 ± 0.8	0.41	0.37	<0.0001
Follow-up diabetic foot care	6.4 ± 1.1	6.3 ± 1.2	6.3 ± 1.2	6.2 ± 1.3	0.69	0.62	0.61
Baseline treatment adherence	6.4 ± 1.9	6.0 ± 2.4	6.3 ± 1.8	5.2 ± 3.1	0.50	0.82	0.16
Follow-up treatment adherence	7.0 ± 0.0	7.0 ± 0.0	7.0 ± 0.0	7.0 ± 0.0	0.98	0.31	0.33

Group 1: no treatment (control group); group 2 treated with α-lipoic acid (800 mg); group 3 treated with α-lipoic acid (100 mg), glutathione (200 mg), and Vitamin D (800 IU); group 4 treated with N-acetyl cysteine (600 mg), glutathione (200 mg), and Vitamin D (800 IU). DN4: Douleur Neuropathique 4 Questions; BPI: Brief Pain Inventory; PAID-5: Problem Areas in Diabetes; SDSCA: Self-Care Diabetes Activities Scale; NRS: Numerical Rating Scales. Student’s t-test for normally distributed data and Mann–Whitney test for not normally distributed numerical data were used for comparison.

**Table 3:** Differences between treatment groups in the changes in questionnaire scores between baseline and follow-up.

	Group 1	Group 2	Group 3	Group 4	*p* Group 1 vs. Group 2	*p* Group 1 vs. Group 3	*p* Group 1 vs. Group 4
BPI severity	−5.1 ± 9.7	−5.1 ± 8.1	−3.8 ± 8.0	−8.0 ± 7.7	0.99	0.60	0.31
BPI interference	−12.5 ± 19.1	−12.2 ± 17.4	−12.1 ± 14.0	−12.7 ± 10.4	0.95	0.94	0.96
NRS	2.2 ± 3.7	−1.6 ± 3.4	−2.3 ± 3.3	−1.7 ± 2.1	0.01	<0.001	<0.001
WHO-5	1.2 ± 7.0	−0.4 ± 7.2	0.3 ± 5.4	0.0 ± 1.2	0.44	0.37	0.44
PAID-5	1.3 ± 4.8	0.7 ± 4.3	1.6 ± 2.6	−0.2 ± 4.0	0.67	0.79	0.27
SDSCA Nutrition	1.1 ± 2.0	1.1 ± 2.2	0.1 ± 1.7	2.7 ± 3.4	0.98	0.07	0.07
SDSCA Physical activity	0.1 ± 3.7	0.1 ± 3.8	0.1 ± 0.8	−0.2 ± 0.4	0.96	0.95	0.68
SDSCA SMBG	0.7 ± 4.2	0.3 ± 3.9	0.4 ± 3.8	−0.2 ± 1.1	0.72	0.77	0.31
SDSCA SMBG adherence	1.6 ± 3.4	1.4 ± 2.7	3.0 ± 3.7	−1.2 ± 2.2	0.86	0.17	0.001
SDSCA Diabetic foot care	3.4 ± 2.8	2.6 ± 2.7	4.0 ± 3.2	0.0 ± 0.7	0.30	0.52	<0.001
SDSCA Treatment adherence	0.6 ± 1.9	1.0 ± 2.4	0.7 ± 1.8	1.7 ± 3.1	0.50	0.82	0.16

Group 1: control group; group 2 treated with α-lipoic acid (800 mg); group 3 treated with α-lipoic acid (100 mg), G = glutathione (200 mg), and Vitamin D (800 IU); group 4 treated with N-acetyl cysteine (600 mg), glutathione (200 mg), and Vitamin D (800 IU). BPI: Brief Pain Inventory; PAID-5: Problem Areas in Diabetes; SDSCA: Self-Care Diabetes Activities Scale; NRS: Numerical Rating Scales. Student’s t-test for normally distributed data and Mann–Whitney test for not normally distributed numerical data were used for comparison.

## Data Availability

The data that support the findings of this study are available from the corresponding author upon reasonable request.
